# Radiogenomic analysis for predicting lymph node metastasis and molecular annotation of radiomic features in pancreatic cancer

**DOI:** 10.1186/s12967-024-05479-y

**Published:** 2024-07-29

**Authors:** Yi Tang, Yi-xi Su, Jin-mei Zheng, Min-ling Zhuo, Qing-fu Qian, Qing-ling Shen, Peng Lin, Zhi-kui Chen

**Affiliations:** 1 Department of Medical Ultrasound, Fujian Medical University Union Hospital, 29 Xinquan road, Fuzhou, China; 2 Department of Radiology, Fujian Medical University Union Hospital, 29 Xinquan road, Fuzhou, China

**Keywords:** Radiomics, Pancreatic cancer, Biological interpretability

## Abstract

**Background:**

To provide a preoperative prediction model for lymph node metastasis in pancreatic cancer patients and provide molecular information of key radiomic features.

**Methods:**

Two cohorts comprising 151 and 54 pancreatic cancer patients were included in the analysis. Radiomic features from the tumor region of interests were extracted by using PyRadiomics software. We used a framework that incorporated 10 machine learning algorithms and generated 77 combinations to construct radiomics-based models for lymph node metastasis prediction. Weighted gene coexpression network analysis (WGCNA) was subsequently performed to determine the relationships between gene expression levels and radiomic features. Molecular pathways enrichment analysis was performed to uncover the underlying molecular features.

**Results:**

Patients in the in-house cohort (mean age, 61.3 years ± 9.6 [SD]; 91 men [60%]) were separated into training (*n* = 105, 70%) and validation (*n* = 46, 30%) cohorts. A total of 1,239 features were extracted and subjected to machine learning algorithms. The 77 radiomic models showed moderate performance for predicting lymph node metastasis, and the combination of the StepGBM and Enet algorithms had the best performance in the training (AUC = 0.84, 95% CI = 0.77–0.91) and validation (AUC = 0.85, 95% CI = 0.73–0.98) cohorts. We determined that 15 features were core variables for lymph node metastasis. Proliferation-related processes may respond to the main molecular alterations underlying these features.

**Conclusions:**

Machine learning-based radiomics could predict the status of lymph node metastasis in pancreatic cancer, which is associated with proliferation-related alterations.

## Background

Pancreatic cancer is one of the most aggressive cancers; it affects more than 400,000 individuals worldwide, and it is estimated that it will be the second most common cause of cancer death in 2040 [1, 2]. It is an aggressive malignancy characterized by a dismal prognosis and high mortality rate, with a mere 10% five-year relative survival rate [3, 4]. Pancreatic ductal adenocarcinoma (PDAC) accounts for approximately 95% of all patients [5]. Although surgery remains the curative option for treating PDAC, its efficacy is limited due to frequent recurrence. Pathologic lymph node (LN) metastasis in PDAC is recognized as a well-established survival indicator [6, 7]. Currently, postoperative histopathology serves as the gold standard for lymph node diagnosis. Unfortunately, routine medical imaging, such as ultrasound (US), computed tomography (CT) and magnetic resonance imaging (MRI), is suboptimal for LN m1etastasis diagnosis [8, 9]. Hence, quantitative analysis of multimodal images is required in the era of precision medicine.

Radiomics, a technique in which features are extracted from medical images, holds great promise for quantifying tumor heterogeneity [10, 11]. Radiomics has provided promising results in predicting pathological, molecular results and clinical outcome in PDAC [12–14]. Recent studies have demonstrated that radiomic features hold promise for predicting LN metastasis [15, 16]. However, reports concerning whether ultrasound-based radiomics could be used for detecting LN metastasis are still limited. Furthermore, the molecular mechanisms underlying these radiomic phenotypes are unclear. Radiogenomics is a technique that infers changes such as gene mutation or expression status from medical images. Several radiogenomic methods for identifying pancreatic cancer have shown exciting value in capturing molecular characteristics [17–19]. The relationships between molecular alterations and radiological findings allow the noninvasive application of medical images for personalized medicine [20]. On the basis of radiogenomic analysis, we could recognize the biological interpretability of radiomics. Therefore, radiogenomics is required for the annotation of clinically applicable radiomic models.

This study aimed to estimate ultrasound-based radiomic model for identifying LN metastasis in patients with PDAC preoperatively. Furthermore, radiogenomic analysis provides novel insights into these radiomic features. This approach provides the possibility of noninvasive diagnosis of LN metastasis in PDAC.

## Methods

### Patients

**Fig. 1:**
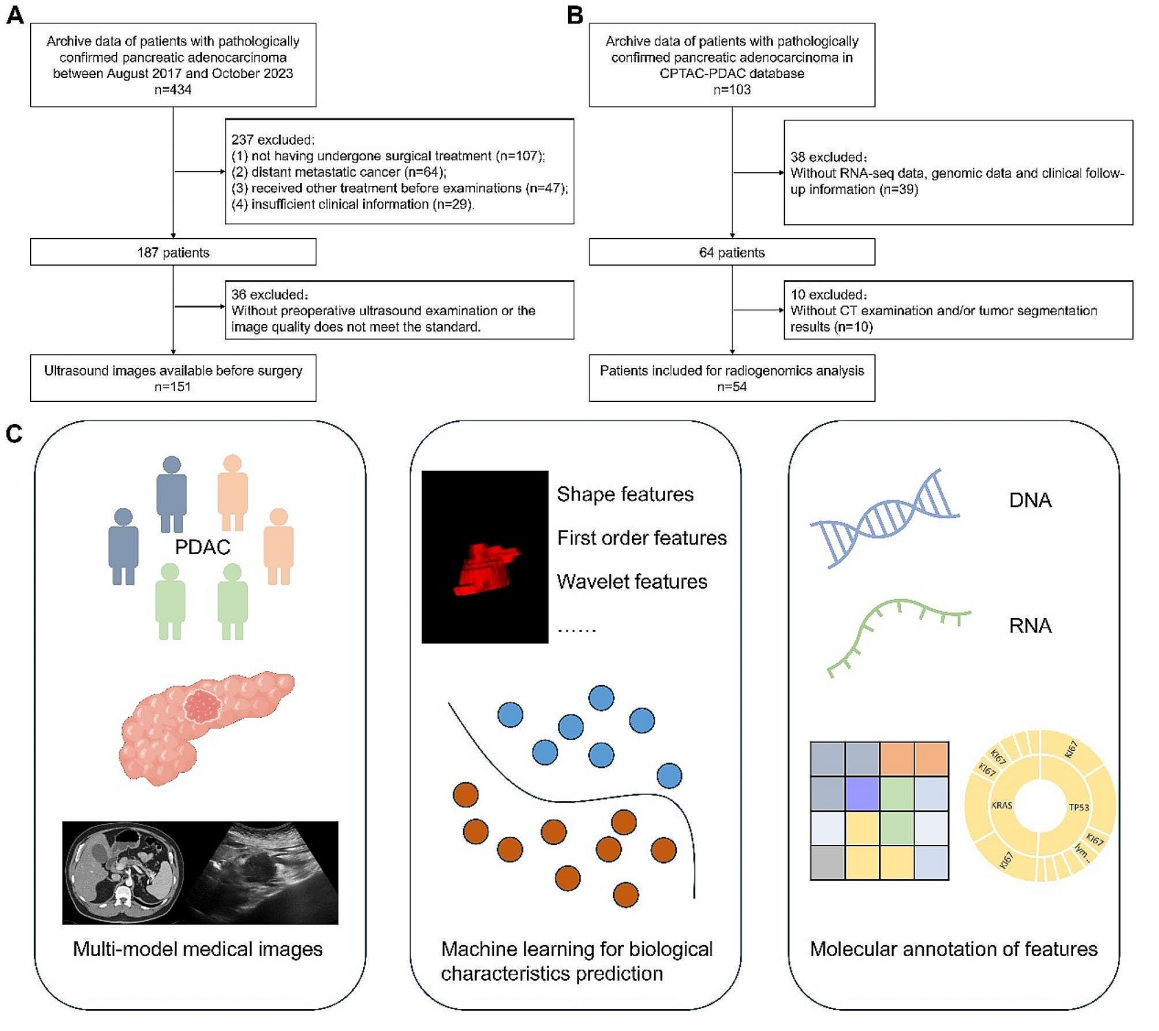
Inclusion flowchart of the study population and study workflow overview. Inclusion of the in-house cohort **(A)** and the CPTAC-PDAC **(B)** cohort. The workflow of this study included multimodal medical image data acquisition, segmentation and radiomic feature extraction, radiomic model development and validation, and radiogenomic analysis for feature annotation

This study was approved by the Institutional Review Board of our hospital, and the requirement for informed consent was waived owing to its retrospective nature. From August 2017 to October 2023, 434 PDAC patients were screened at Fujian Medical University Union Hospital. Finally, 151 patients were eligible and included in our study. Patients who satisfied all of the following criteria were included: (a) diagnosis of resectable PDAC; (b) a history of standard LN dissection performed during the operation and whose ≥ 16 lymph nodes were removed; and (c) all PDAC patients undergone ultrasound examination within 4 weeks before surgery. The exclusion criteria were as follows: (a) pathological results obtained only by biopsy puncture; (b) distant metastasis of the tumor; (c) any history of preoperative chemotherapy and/or chemoradiotherapy; (d) lack of sufficient clinical information, including lymph node metastasis status; and (d) lacked of clear or not obvious ultrasound images. Clinical data, including age, sex, tumor size, histological grade, LN metastasis status and CA19-9 concentration, were collected from the patients’ electronic medical records. Furthermore, CT images and corresponding molecular information from a total of 54 patients in the CPTAC-PDAC cohort [21] were obtained from the Cancer Imaging Archive (TCIA) database [22, 23] for radiogenomic analysis. The detailed patient inclusion criteria are summarized in Fig. 1.

### Tumor segmentation and radiomics feature extraction

**Fig. 2:**
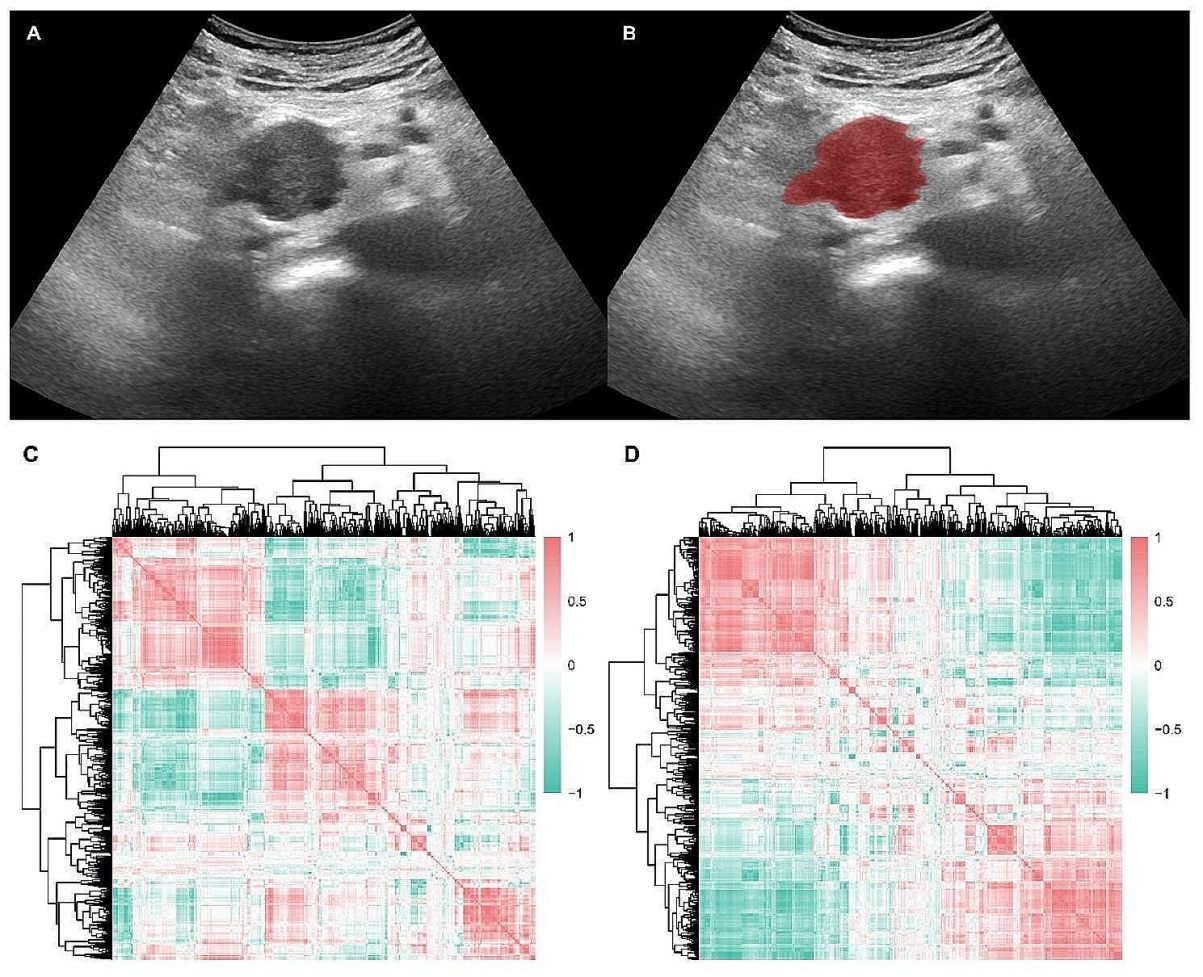
Radiomic features extracted from the in-house cohort. **(A)** A 59-year-old male patient with pancreatic cancer; **(B)** lesions were segmented manually. Radiomic features were extracted from the training **(C)** and validation **(D)** cohorts and Spearman correlation analyses suggested that radiomic features have internal correlations and heterogeneity

For the in-house cohort, the regions of interest (ROIs) of the tumor areas were manually delineated on the basis of grayscale images generated by an experienced radiologist via ITK-SNAP software (version 4.0.1) [24] (Fig. 2). For the CPTAC-PDAC cohort, segmentation files of tumors were obtained from the annotated imaging package RTSTRUCT from the TCIA. Radiomic features were extracted from the ROIs. A total of 1,239 radiomic features, including first-order, shape and texture features, were extracted by using PyRadiomics software (version 3.0.1) [25]. The textural features are subdivided into the following classes: (1) gray-level co-occurrence matrix (GLCM), (2) gray-level run-length matrix (GLRLM), (3) gray-level size zone matrix (GLSZM), (4) neighborhood gray-tone difference matrix (NGTDM), and (5) gray-level dependence matrix (GLDM) features. Several filters, including exponential, logarithm, square, square root and wavelet, were also utilized for feature extraction.

### Feature selection and model construction

The in-house cohort was subsequently randomly assigned to the training and validation cohort at a ratio of 7:3. Prior to analysis, the radiomic features were standardized using the Z-score algorithm. In the training cohort, Wilcoxon analysis was performed. Then, to develop a radiomic model, we integrated 10 types of machine learning and 113 algorithm combinations. And models that included more than five features were included. The ten machine learning algorithms used were as follows: SVM, glmBoost, Ridge, Lasso, Enet, Stepglm, GBM, LDA, XGBoost and naive Bayes. For each model, the area under the receiver operating characteristic curve (AUC) was calculated, and the machine learning algorithm with the highest average AUC was considered optimal. Then, the sensitivity, specificity, negative predictive value and positive predictive value were calculated to compare the performance of the different models.

### Radiogenomic analysis

Transcriptomic and clinical data were downloaded from the CPTAC pan-cancer project. Weighted correlation network analysis (WGCNA) was performed to determine the relationships between the gene modules and radiomic features. Only messenger RNAs (mRNAs) were subjected to WGCNA. The adjacency matrix was created using a soft threshold of 7. Next, a topological overlap matrix (TOM) was constructed using a hierarchical clustering dendrogram to delineate distinct modules based on similar gene expression. WGCNA was performed based on the following parameters: power = 7, minModuleSize = 30, and mergeCutHeight = 0.3. Finally, we identified the module eigengene expression profiles to examine the relationship between the modules and radiomic features.

The genes involved in each module were subjected to gene set enrichment analysis. Kyoto Encyclopedia of Genes and Genomes (KEGG) pathway enrichment was determined by using the DAVID online tool [26].

## Results

### Patient characteristics

A total of 151 patients (91 men, 60 women) with a mean age of 61.3 ± 9.6 years (SD) were included in the lymph node metastasis prediction analysis. Furthermore, 54 patients (25 men, 29 women) with a mean age of 60.1 ± 10.9 years (SD) were included in the radiogenomic analysis. The clinicopathologic characteristics of the patients included are summarized in Table 1. For the in-house cohort, patients were separated into training (*n* = 105) and validation (*n* = 46) cohorts at a ratio of 7:3. There were no significant differences in clinicopathological features between the two subgroups (Table 1).

**Table 1:** Patients’ characteristics

Characteristic		In-house cohort (*n* = 151)			*P* value	CPTAC-PDAC(*n* = 54)
		All	Training (*n* = 105)	Validation (*n* = 46)		
Age (y) *		62 (55-68.5)	61 (55–68)	64 (57-70.25)	0.09	65.5 (62-71.75)
**Sex**						
	Male	91 (60.3)	60 (57.1)	31 (67.4)	0.236	25 (46.3)
	Female	60 (39.7)	45 (42.9)	15 (32.6)		29 (53.7)
Tumor size*		3.6 (2.9–4.2)	3.6 (2.9–4.1)	3.4 (2.9–4.5)	0.808	3.9 (2.9–4.8)
**Histological grade**						
	Well or moderate	108 (71.5)	79 (75.2)	29 (63.0)	0.355	39 (72.2)
	Poor	28 (18.5)	18 (17.1)	10 (21.7)		15 (27.8)
	NA	15 (9.9)	8 (7.6)	7 (15.2)		-
**Lymph node metastasis**						
Positive		82 (54.3)	56 (53.3)	26 (56.5)	0.717	45 (83.3)
Negative		69 (45.7)	49 (46.7)	20 (43.5)		7 (13.0)
NA		-	-	-		2 (3.7)
**CA 19 − 9 (U/ml)**						
	≤ 300	97 (64.2)	68 (64.8)	29 (63.0)	0.839	-
	> 300	54 (35.8)	37 (35.2)	17 (37.0)		-

*Data are medians, with IQRs in parentheses

**Table 2:** KEGG pathways enriched by genes in blue and magenta modules

Term	Count	Ratio (%)	*P*-Value
**Blue module**			
hsa04110: Cell cycle	44	6.48	7.39E-24
hsa04115: p53 signaling pathway	17	2.50	5.53E-08
hsa03030: DNA replication	10	1.47	1.36E-05
hsa03460: Fanconi anemia pathway	11	1.62	7.44E-05
hsa05222: Small cell lung cancer	14	2.06	1.23E-04
hsa04114: Oocyte meiosis	17	2.50	1.25E-04
hsa04814: Motor proteins	21	3.09	1.89E-04
hsa04210: Apoptosis	17	2.50	1.94E-04
hsa05230: Central carbon metabolism in cancer	11	1.62	6.75E-04
**Magenta module**			
hsa01100: Metabolic pathways	29	17.37	2.81E-04
hsa05204: Chemical carcinogenesis - DNA adducts	5	2.99	0.004
hsa00982: Drug metabolism - cytochrome P450	5	2.99	0.005
hsa00590: Arachidonic acid metabolism	4	2.40	0.020
hsa04927: Cortisol synthesis and secretion	4	2.40	0.023
hsa00830: Retinol metabolism	4	2.40	0.026
hsa04360: Axon guidance	6	3.59	0.029
hsa00591: Linoleic acid metabolism	3	1.80	0.032
hsa04918: Thyroid hormone synthesis	4	2.40	0.034

### Machine learning model development

From the tumor ROIs, we extracted 1,239 features from the training and validation cohorts (Fig. 2A-B). The features in the training (Fig. 2C) and validation (Fig. 2D) cohorts were Z scores for further analysis. These features showed great heterogeneity, and some features implemented tight clustering.

**Fig. 3:**
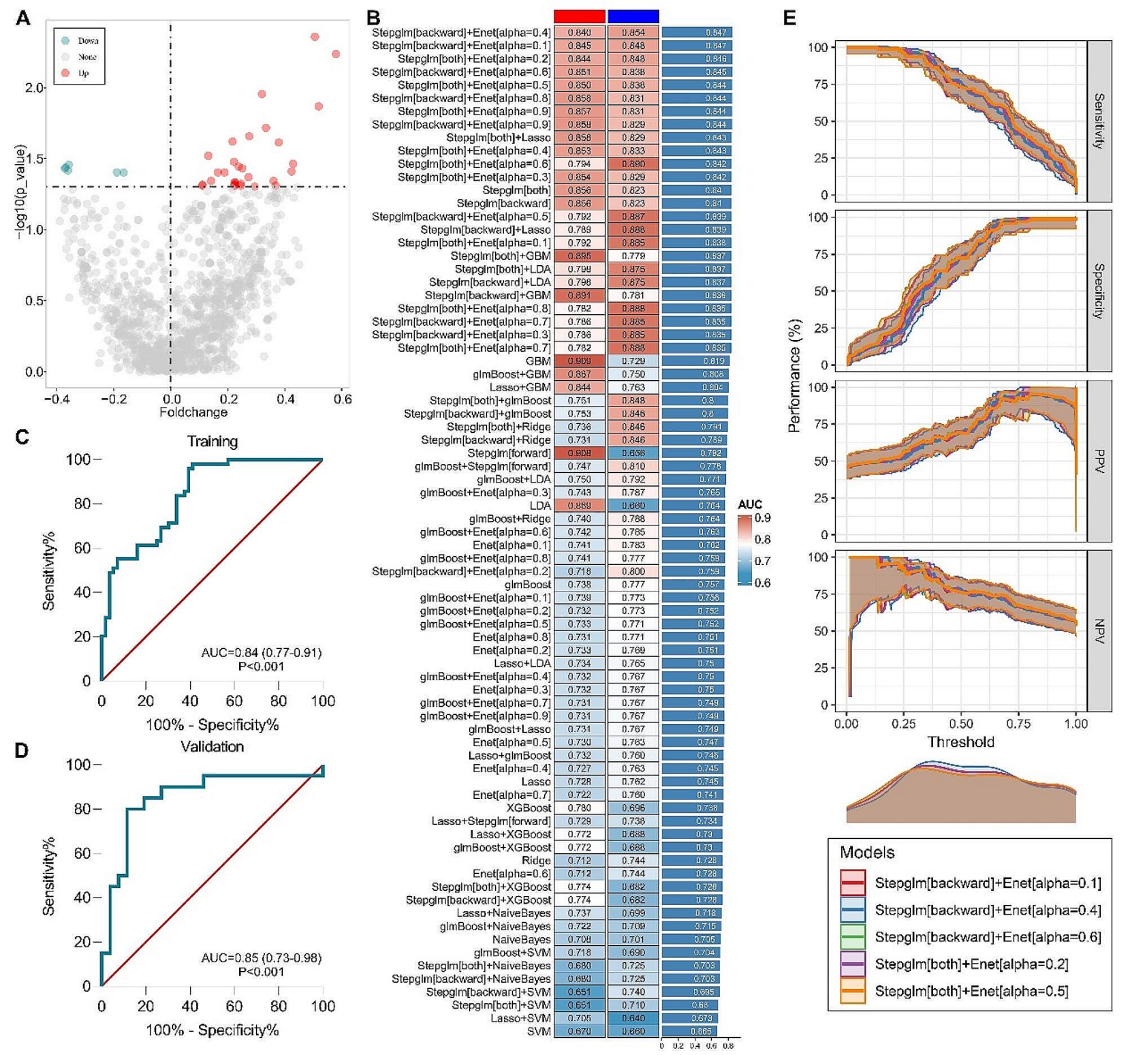
Model development and validation for lymph node metastasis. **(A)** Volcano plot showing radiomic features that are differentially expressed between patients with different lymph node metastasis statuses. **(B)** A total of 77 combinations of machine learning algorithms were used for the LN metastasis prediction models. The AUC values for the training and validation cohorts and AUC of each model were calculated. ROC curves for the training **(C)** and validation **(D)** cohorts for the optimal machine learning model. **(E)** Sensitivity, specificity, positive predictive value, and negative predictive value of the five optimal models

Based on the radiomic feature profiles of patients in the training cohort, the Wilcoxon test identified 37 differentially expressed radiomic features in the training cohort (Fig. 3A). These 37 features were subsequently subjected to our machine learning-based prediction model. Then, we fitted 77 kinds of prediction models with more than 5 features and calculated the AUC of each model (Fig. 3B). The optimal model was a combination of Stepglm (direction = backward) and elastic net (alpha = 0.4) with the highest average AUC (0.847). The AUCs in the training and validation cohorts were 0.84 (95% CI: 0.77–0.91) (Fig. 3C) and 0.85 (95% CI: 0.73–0.98), respectively (Fig. 3D). Furthermore, other machine learning models also exhibited moderate performance for the prediction of lymph node metastasis. Model performance was calculated and used for comparison. The consistency of the results of multiple models indicated the stability of the discrimination efficiency. The parameters reflecting the prediction performance of the top five models demonstrate a very good consistency (Fig. 3E).

### Relationships between radiomic features and gene expression

**Fig. 4:**
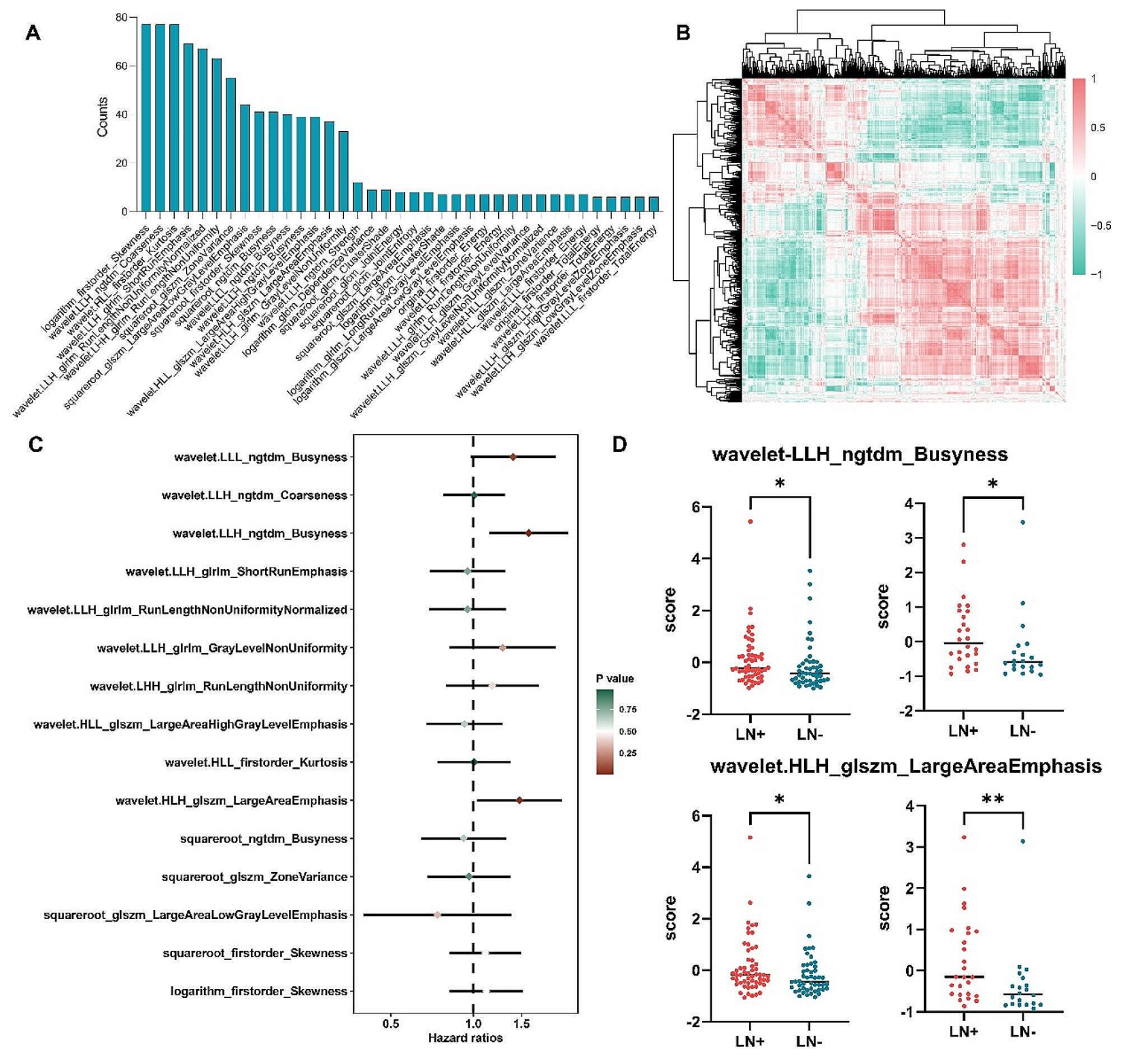
Determination of lymph node (LN) metastasis-specific radiomic features and their prognostic value. **(A)** The incidence of specific radiomic features included in 77 distinct prognostic models. **(B)** A heatmap of radiomic features derived from the CPTAC-PDAC cohort. **(C)** Univariate Cox regression analyses show the associations between overall survival (OS) and the 15 most crucial LN metastasis-related features. **(D)** A high score of the wavelet-LLH_ngtdm_Busyness feature and the wavelet-HLH_glszm_LargeAreaEmphasis feature correlate not only with poorer OS but also with the presence of LN metastasis

In the process of machine learning algorithms, 15 radiomic features were determined to be key features because they were incorporated into more than 30 models of 77 machine learning algorithms (Fig. 4A). To achieve molecular annotation of these features, radiomic features were extracted from the CPTAC-PDAC cohort (Fig. 4B). To further determine the prognostic values of the 15 features, we utilized univariate Cox analysis and found high score of wavelet-LLH_ngtdm_Busyness feature and the wavelet-HLH_glszm_LargeAreaEmphasis were significant related to inferior OS (Fig. 4C). In in-house cohort, high score of the two features were also observed in lymph node metastasis patients (Fig. 4D).

**Fig. 5:**
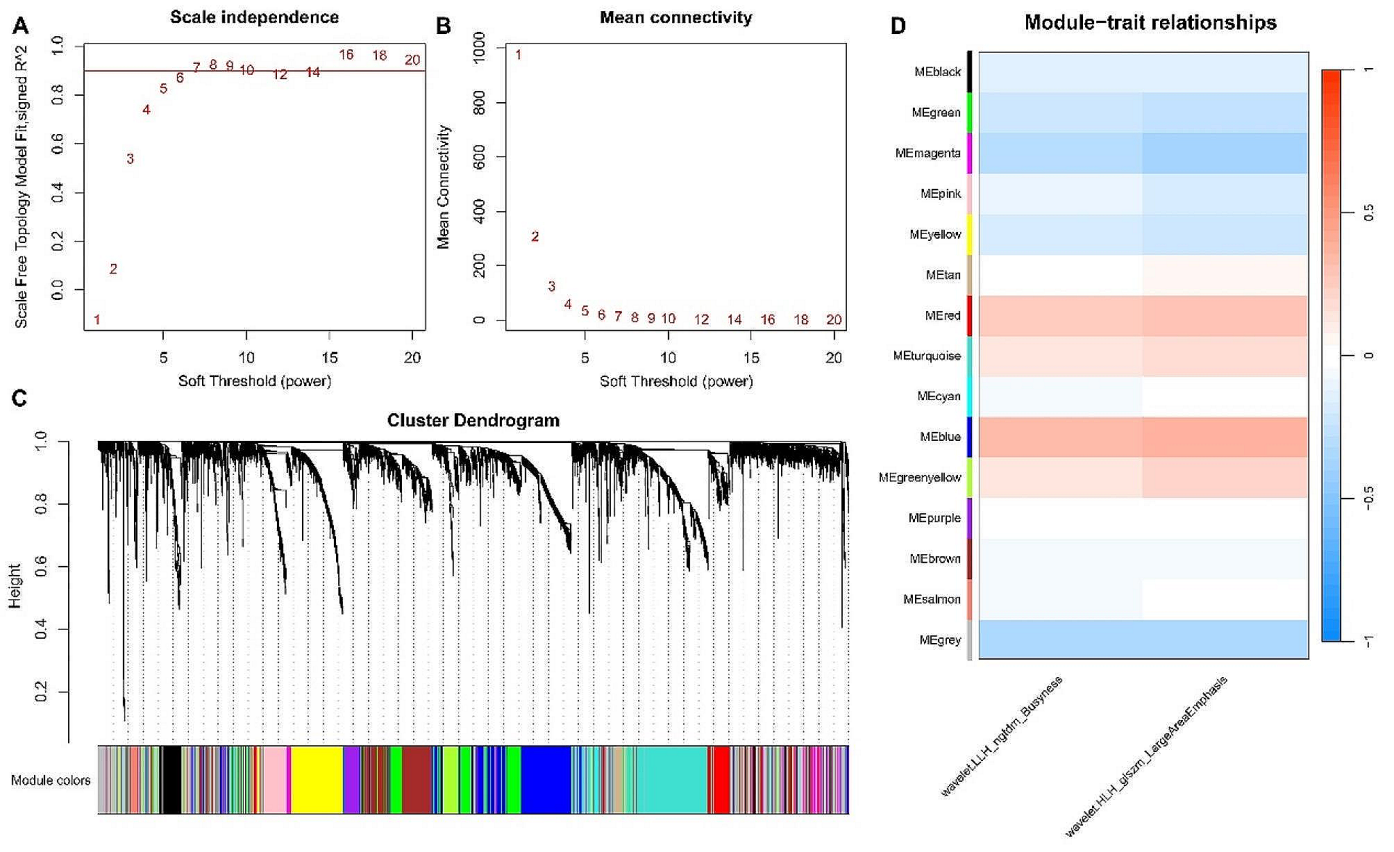
Identification of relationships between LN metastasis-specific radiomic features and gene modules. **(A-B)** Soft threshold determination; **(C)** Module detection by gene cluster dendrograms; **(D)** Module-trait associations revealed by the Pearson correlation coefficient

Then, WGCNA was performed to construct a radiogenomic map. For the WGCNA of the radiogenomic datasets, the soft-thresholding power was 7, and the mean connectivity was also stable when the soft-thresholding power was set to 7 (Fig. 5A-B). A hierarchical clustering tree showed that 15 gene modules had clustered (Fig. 5C). Then, wavelet-LLH_ngtdm_Busyness feature and the wavelet-HLH_glszm_LargeAreaEmphasis were submitted to determine their correlations with the 15 modules (Fig. 5D). The blue module exhibits a significant positive correlation with the two features, while the magenta module shows a significant negative correlation with them.

**Fig. 6:**
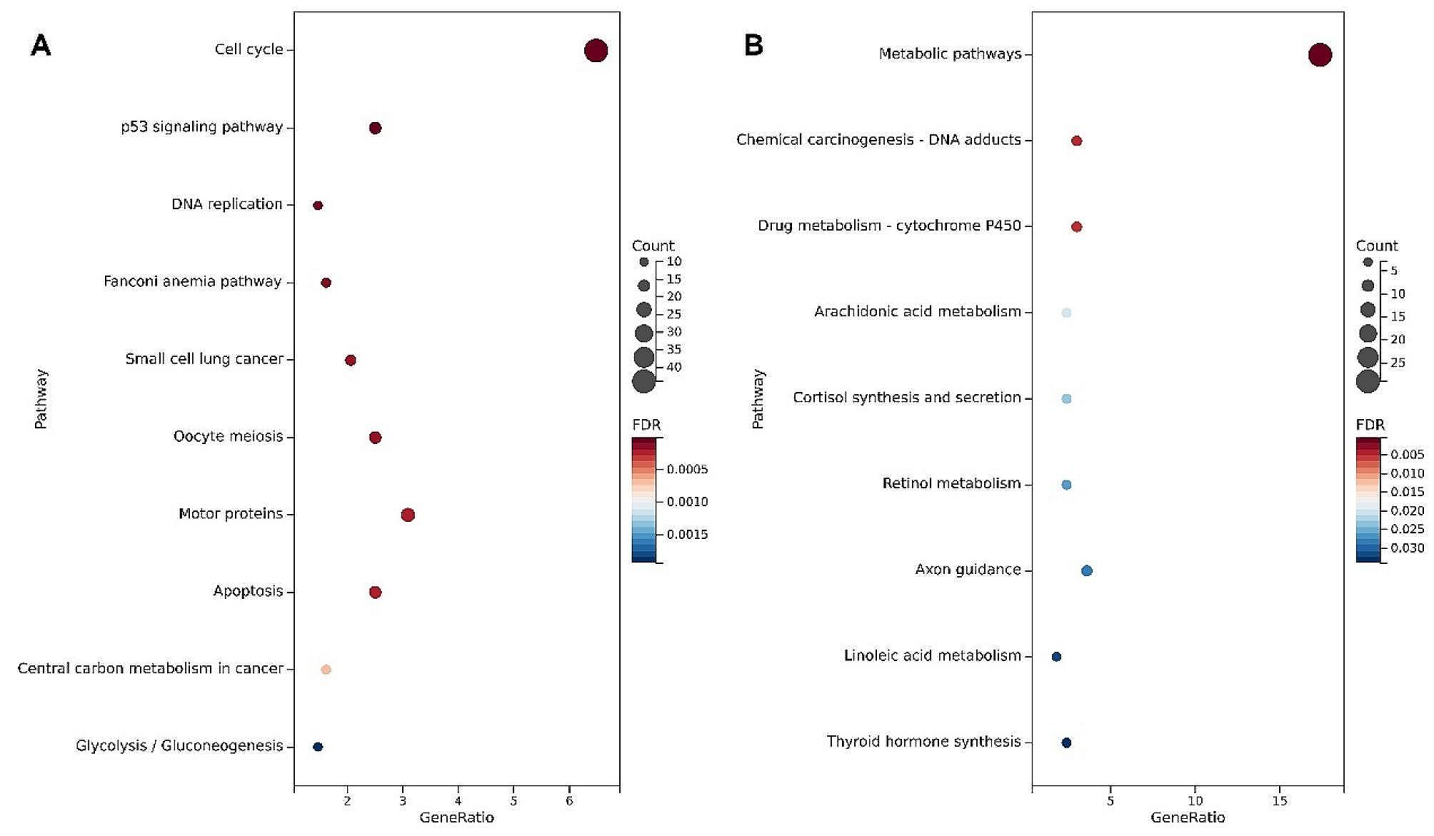
KEGG pathway enrichment analysis for each gene module. The top 10 most significant KEGG pathways for **(A)** blue and **(B)** magenta

The genes involved in each module were subjected to gene set enrichment analysis. The different gene modules represented different molecular processes (Table 2). The top three KEGG pathways enriched in the blue module-related genes were “Cell cycle”, “p53 signaling pathway”, “DNA replication”. ” (Fig. 6A). For the magenta module, “Metabolic pathways”, “hsa05204: Chemical carcinogenesis - DNA adducts”, “Drug metabolism - cytochrome P450” were most significantly enriched (Fig. 6B).

## Discussion

Individualized multi-omics data are needed to provide tailored medical intervention plans for precision medicine [27, 28]. Here, we investigated the performance of radiomics for the prediction of LN metastasis prediction in PDAC patients. Furthermore, radiogenomic analysis provided molecular information on key radiomic features. This study explored the value of radiomics in evaluating the biological behavior of pancreatic cancer patients and provided ideas for the specific biological interpretation of radiomic features.

PDAC is an aggressive tumor type, and LN metastasis is an independent predictor of PDAC survival [29, 30]. A prior investigation involving 3,478 patients revealed that 1,971 (56.7%) presented with lymph node metastasis. Consequently, PDAC patients with lymph node metastasis experienced reduced OS compared to those without such metastasis [31]. The incidence of PDAC lymph node metastasis observed in this study aligns closely with our own findings. For most tumors, LN metastasis should be diagnosed based on pathological results. However, preoperative pathological results are still difficult to obtain owing to the anatomical position of the pancreas. Furthermore, biopsy results may also be false-negatives. Although many previous studies have determined some risk stratification approaches for LN metastasis, the preoperative determination of LN metastasis status still depends on radiological examination [32]. Hence, several previous studies have proposed the use of radiomics-based medical image analysis for LN metastasis prediction. For example, Zeng et al. compared the performance of CT and MRI radiomic models for predicting LN metastasis in PDAC and reported that an MRI-based radiomic model may provide superior predictive performance when compared with CT-based radiomic data [33]. Another study showed that an artificial intelligence model outperformed radiomic models for the prediction of LN metastasis [34]. Ultrasound-based radiomic data have also demonstrated the effectiveness of accurately predicting personalized pathological tumor molecular features. Several studies utilized ultrasound-based radiomic analysis for LN metastasis in different cancer types, including breast, thyroid and tongue cancer [35–37]. We found that ultrasound-based radiomics also exhibited moderate performance in predicting LN metastasis preoperatively. Interestingly, endoscopic ultrasonography has gradually become one of the main examinations used to detect pancreatic diseases [38]. The role of ultrasound-based radiomics should be further determined.

Radiogenomic analysis revealed relationships between molecular alterations and radiomic features [39]. To date, several studies have explored the correlation between radiomic features and gene expression profiles in patients with various malignancies, especially lung, breast and brain cancers. For example, several studies have explored the performance of radiomics for epidermal growth factor receptor (EGFR) mutation status prediction in lung cancer [40–42]. Radiogenomic analyses have also been applied to analyze the associations between radiomic features and biological functions, such as HER2 expression in breast cancer [43, 44]. The integration of radiomic features and RNA-seq data should be explored to provide molecular information for computational algorithms. The utility of radiomic features in PDAC should be explored across diverse research objectives. Multiple other studies have investigated the gene expression profiles of pancreatic cancer and radiomic features in PDAC [19, 45]. In our study, WGCNA was performed to determine the gene modules that correlated with key LN metastasis radiomic features. We found that many molecular processes are key processes that are responsible for these features and are used for LN metastasis prediction. For example, proliferation-related pathways were significantly related to features for LN metastasis prediction. This also explains why these features are included in the predictive model for lymph node metastasis. However, the underlying specific molecular mechanisms still need to be analyzed.

This study has several limitations. First, the limited sample size may influence the external validity and generality of our findings to different populations. Therefore, future larger, multicenter, prospective studies will be critical to validate our findings. Second, it is also important to note that combining different medical image models could lead to cross-modal discovery and enhance the robustness of our analysis. Our study included two medical image models, US and CT, which may influence the stability of the results. However, future studies across different imaging modes are necessary to determine the clinical applicability of these key features. Third, although radiogenomic analysis can reveal correlations between radiomic features and molecular information, further investigations of their interrelationships are needed.

## Conclusions

In conclusion, we verified a novel radiomic predictive model that has moderate performance for identifying pancreatic cancer-related lymph node metastasis. Furthermore, we determined the molecular alterations associated with these features. Radiogenomics may help both precision and personalized medicine.

## Data Availability

The data and materials used to support the findings of this study are available from the corresponding author upon request.

## Abbreviations

CT: Computed tomography
EGFR: Epidermal growth factor receptor
GO: Gene ontology
KEGG: Kyoto Encyclopedia of Genes and Genomes
MRI: Magnetic resonance imaging
PDAC: Pancreatic ductal adenocarcinoma
ROC: Receiver operating characteristic curve
RTSTRUCT: Radiotherapy structure set
US: Ultrasound
WGCNA: Weighted gene coexpression network analysis
